# Establishing a Canadian national clinical trials network for kidney disease: proceedings of a planning workshop

**DOI:** 10.1186/s40697-015-0080-7

**Published:** 2015-11-17

**Authors:** Claudio Rigatto, Michael Walsh, Nadia Zalunardo, Catherine M Clase, Braden J Manns, François Madore, Susan M Samuel, Catherine J Morgan, Wim Wolfs, Rita S. Suri

**Affiliations:** Department of Medicine, University of Manitoba, Winnipeg, MB Canada; Department of Medicine, McMaster University, Hamilton, ON Canada; Department of Medicine, University of British Columbia, Vancouver, BC Canada; Department of Medicine, University of Calgary, Calgary, AB Canada; Department of Community Health Sciences, University of Calgary, Calgary, AB Canada; Centre de Recherche, Hôpital du Sacré-Cœur de Montréal, University de Montréal, Montreal, QC Canada; Department of Pediatrics, University of Calgary, Calgary, AB Canada; Department of Pediatrics, University of Alberta, Edmonton, AB Canada; Kidney Foundation of Canada, Montreal, QC Canada; Centre de Recherche, Centre Hospitalier de l’Université de Montréal, University de Montréal, Montreal, QC Canada; Section de Néphrologie, Centre Hospitalier de l’Université de Montréal, University of Montreal, 1058 rue St. Denis, Montreal, QC H2X 3J4 Canada

## Abstract

Knowledge generation through randomized controlled trials (RCTs) is critical to advance the medical evidence base, inform decision-making, and improve care and outcomes. Unfortunately, nephrology has typically lagged behind other medical specialties in this regard. The establishment of formal clinical trial networks can facilitate the successful conduct of RCTs and has significantly increased the number of RCTs performed worldwide in other medical specialties. No such formal network of nephrology trialists exists in Canada. On April 24, 2014, the Canadian Kidney Knowledge Translation and Generation Network (CANN-NET) Clinical Trials Committee held a stakeholder engagement meeting to address this gap and improve the nephrology clinical trial landscape in Canada. The meeting was held in Vancouver in association with the 2014 Canadian Society of Nephrology Annual General Meeting and was co-sponsored by the Kidney Foundation of Canada and CANN-NET. Attendees included nephrologists from university- and non-university-affiliated nephrology practices, administrators, and representatives from the Kidney Foundation of Canada. Through structured presentations and facilitated group discussions, the group explored the extent to which nephrology trials are currently happening in Canada, barriers to leading or participating in larger investigator-initiated trials, and strategies to improve clinical trial output in nephrology in Canada. The themes and action items arising from this meeting are discussed.

**Electronic supplementary material:**

The online version of this article (doi:10.1186/s40697-015-0080-7) contains supplementary material, which is available to authorized users.

## Abrégé

La création d’un bagage de connaissances commun par la conduite d’essais cliniques est essentielle pour assurer l’avancement des notions de base en médecine, étayer la prise de décisions, améliorer les soins aux patients et assurer de meilleurs résultats cliniques. L’établissement d’un réseau officiel et reconnu de partage des connaissances issues d’essais cliniques peut en faciliter la conduite et assurer leur bon déroulement. La preuve en est faite par l’augmentation du nombre d’essais cliniques probants ayant été menés à travers le monde, dans d’autres disciplines médicales. Malheureusement, la néphrologie tire de l’arrière à cet égard par rapport aux autres spécialités, un tel réseau de partage n’existe pas dans le domaine au Canada. C’est dans ce contexte que le 24 avril 2014, le comité des essais cliniques de la « Canadian Kidney Knowledge Translation and Generation Network » (CANN-NET) a tenu une assemblée générale afin de mobiliser les parties intéressées. On a voulu leur exposer cette lacune, tenter d’apporter des solutions et ultimement, faire progresser le bilan de la néphrologie en cette matière. La conférence, en collaboration avec l’assemblée générale annuelle de la Société canadienne de néphrologie, s’est tenue à Vancouver et était subventionnée conjointement par la Fondation canadienne du Rein et le CANN-NET. Parmi les participants, on comptait des néphrologues pratiquants associés ou non à un établissement universitaire ainsi que des administrateurs et des représentants de la Fondation canadienne du Rein. À l’aide de présentations structurées et de discussions de groupe, les participants ont pu observer l’état actuel des essais cliniques au pays, identifier les barrières entravant la participation à plus grande échelle à des essais entrepris sous l’initiative d’un chercheur, et discuter de stratégies pour améliorer les résultats d’essais cliniques en néphrologie au Canada. Le présent article fait état des thèmes abordés lors de cette assemblée et des mesures à prendre pour atteindre les objectifs fixés.

## Overview

### Context

Knowledge generation through randomized controlled trials (RCTs) is critical to advance the medical evidence base, inform decision-making, and improve care and outcomes. The Canadian Kidney Knowledge Translation and Generation Network (CANN-NET) was established in 2010 as a partnership organization that links Canadian kidney disease guideline producers, knowledge translation specialists, and knowledge users to improve knowledge dissemination and care of patients with kidney disease. CANN-NET brings together a national group of experienced researchers to address knowledge gaps in the treatment and management of kidney disease in Canada. On April 24, 2014, the CANN-NET Clinical Trials Committee held a stakeholder engagement meeting aimed at establishing a formal Canadian Nephrology Trials Network in order to improve the relevance, number, and quality of nephrology clinical trials in Canada. The meeting was held in Vancouver in association with the 2014 Canadian Society of Nephrology Annual General Meeting and was co-sponsored by the Kidney Foundation of Canada and CANN-NET. Attendees included nephrologists from university- and non-university-affiliated nephrology practices, administrators, and representatives from the Kidney Foundation [1]. Through structured presentations and facilitated group discussions, the group explored the extent to which nephrology trials are currently happening in Canada, barriers to leading or participating in larger investigator-initiated trials, and strategies to improve clinical trial output in nephrology in Canada.

### Suggested goals for a Canadian clinical trial network for kidney disease

While it was recognized that many Canadian renal investigators are doing excellent work and running clinical trials through informal networks, there was enormous enthusiasm for, and endorsement of, creating a more formal Canadian nephrology clinical trial network. Suggested goals of this network were as follows:

- To promote a culture of collaboration rather than competition within the Canadian nephrology community
- To promote ongoing discussion of clinical trial ideas in order to develop new studies
- To identify and address barriers to recruitment for ongoing studies
- To hold regular face-to-face meetings in order to accomplish the first three goals.
- To develop a web-based system to connect investigators with interested centers across Canada
- To develop a central infrastructure of methodologists and coordinators to assist motivated investigators in the development and conduct of multicenter clinical trials
- To promote, prioritize, and facilitate funding of nephrology RCTs through review and endorsement of protocols and create national and local advocacy for funding high-priority studies

### Barriers and solutions

Barriers to the conduct of RCTs and potential solutions were explored (Table 1). Key barriers identified were as follows:

**Table 1:** Barriers and potential solutions to conducting nephrology trials in Canada

Specific barrier	Potential solutions
Lack of engagement at community sites	
♦ MD engagement • Site PIs may have no vested interest • Multiple physicians in a shared care clinic model: may not have buy-in from all treating MDs	⇒ Involve community centers and site PIs earlier in the process (i.e., during protocol development) to get “buy-in”; learn local practices and pitfalls early ⇒ Identify local champions (mentioned repeatedly)—MDs, nurses, allied health, and patients
♦ Nursing engagement • Treating nurses not engaged	⇒ Involve local nurses and allied health in steering committee to get buy-in ⇒ CANN-NET Clinical Trials Committee could assist in developing the skill set of local champions
♦ Patient engagement	⇒ Advertise studies to patients better ⇒ See below
♦ Inability to sustain momentum: physicians are busy and the ongoing commitment, time, and effort required to continue participation is often too high	⇒ Simplify protocols so that minimal time is required (autopilot study) ⇒ Increase role of central/site coordinators to automate management
♦ Lack of communication between PI and local centers	⇒ Increase PI presence at the community sites and provide feedback on recruitment success and deliverables—periodic newsletters, recruitment progress tables, personal phone calls, site visits ⇒ It was emphasized that this should not just be emails
♦ Lack of trained research nurses or coordinators • Not enough work to maintain a full-time research coordinator • Some nurses willing to do part-time RCT work but do not have proper training • Lack of financial support for research nurses	⇒ Provide CANN-NET central coordinator who could • Provide training/support for part-time personnel • Assist with ethics ⇒ Simplify protocols to reduce workload—decrease follow-up visits, data collection, etc. ⇒ Coordinator from academic site could recruit patients at community centers if distances are not too far—grants should thus budget for travel; facilitate through CANN-NET ⇒ Provide more funding (via grant) to allow research nurse salaries to be in line with clinical salaries ⇒ Hire people on a lower pay-scale for tasks not requiring advanced skill set, e.g., data entry
Lack of engagement of patients (at all sites)	
♦ Patients feel trials are a burden; they may feel it is a disruption to their care	⇒ Present trials as an option for patients to improve their care (similar to the way oncology trials are presented) rather than giving perception that patients are doing investigators a favor ⇒ Engage patients directly through advertising ⇒ Engage patients during protocol development stage ⇒ Conduct focus groups to determine what the barriers are to patient participation; facilitate through CANN-NET ⇒ Get local buy-in from nurses and allied health
♦ Patients are “trialed out”—same populations for different trials means same patients are being asked again and again	⇒ See below
♦ Cognitive and language barriers	⇒ Understand the impact of these at the local level ⇒ Simplify and translate consent forms ⇒ Central training of coordinators to improve comprehension of trial participation
Competition and overlap	
♦ Too many trials in overlapping populations; competition with other trials	⇒ Local sites could state interests and concentrate participation on a few trials at a given time ⇒ CANN-NET Clinical Trials Committee could assist in matching the right project with the right site (patient population) through web-based registry ⇒ Engage more community sites that are not participating in any trials as yet through CANN-NET
Onerous Research Ethics Board requirements	
♦ Separate REB for each site is time and effort consuming	⇒ CANN-NET should • Advocate for a national REB standard • Advocate for an expedited site review process for protocols approved at a central site
Language and cultural barriers	
♦ French sites often left out of trials for this reason and this is a lost opportunity ♦ Limited communication between investigators in and outside Quebec	⇒ This barrier is often artificial (perceived rather than real) as trial-related materials are often in multiple languages including French; improved communication with centers would assist with this problem ⇒ Trial budgets should include money for translation of materials; this cost is justified by the importance of including Canadians from diverse backgrounds and the increased potential for recruitment ⇒ CANN-NET could assist with translation ⇒ Increase communication, networking, and collaboration between Quebec and other provinces via CANN-NET
Lack of funding for and prioritization of nephrology trials	
	⇒ Need to increase exposure of importance of renal disease to provincial agencies; e.g., could CANN-NET convince provincial renal agencies to match CIHR/KFoC funding for certain successful grants addressing network priorities? ⇒ National agencies and this CANN-NET network could align efforts to improve branding (advertising, advocates) in order to increase exposure of nephrology disease and nephrology research and secure more funding

*PI* principal investigator, *MD* medical doctors, *RC* research coordinator

1. *Lack of engagement and core expertise at community sites*. Active engagement, training, and support of local champions of clinical studies, particularly in centers that are not affiliated with a university, was stressed as a key part of the solution, as was the provision of centralized research coordination services to assist smaller centers with conducting a clinical trial (detailed in Table 1).
2. *Lack of patient engagement at all levels of trial development and conduct*. A multifaceted patient engagement strategy aimed at promoting a culture of participation among patients was proposed and is summarized in Table 1.
3. *Competition for recruitment and overlap between trials*. Strategies to increase the pool of participating centers and to better match trials with participating centers were discussed.
4. *Onerous Research Ethics Board requirements*. It was suggested that CANN-NET advocate for a national harmonized REB standard and expedited local site review for protocols already approved at other sites.
5. *Language and cultural barriers*. This issue is intrinsic to a multicultural and multilingual society, and various solutions were proposed and summarized in Table 1.
6. *Lack of funding for, and prioritization of, nephrology trials*. The importance of advocacy to increase funding and in-kind support for RCTs in nephrology at local and national levels was stressed as a key component of the solution.

### Specific action items

Based on the themes elicited at the meeting, the following specific action items were endorsed by the CANN-NET Clinical Trials Committee:

1. The committee will proceed with the formation of a Canadian clinical trial network, planning twice-yearly meetings of network participants to be held initially in conjunction with national or international society meetings.
2. The committee will engage with the Kidney Foundation of Canada and other agencies as needed to develop online networking tools as described.
3. The committee will engage with the Kidney Foundation of Canada to launch a new funding competition for meeting and planning grants with the intent of providing financial support for meetings that will enable the conduct of prospective research (i.e., randomized controlled trials or prospective cohort studies).
4. The committee will further consider ways in which a core RCT infrastructure might be funded and maintained.
5. The committee will advocate for a more patient-centered approach to nephrology research by supporting studies that include patient- and/or caregiver-identified priorities.

## Logistics

### Planning and funding

A meeting subcommittee (RSS, MW, CR, BJM, WW) planned the meeting, set the agenda, and facilitated the discussion. The meeting was scheduled to coincide with the 2014 Canadian Society of Nephrology (CSN) annual meeting, on April 24, 2014. Funding was provided by the Kidney Foundation of Canada,

### Invitees

It was recognized that the inclusion of patients and centers representative of diverse Canadian practice contexts would be critically important to any future network, and that community and academic health care practitioners alike benefit from improving the nephrology evidence base. We thus invited representatives from centers with and without university affiliations. The meeting co-chairs wrote to the heads of all 15 Canadian university-affiliated nephrology divisions, and to 33 non-university-affiliated service chiefs, explaining the nature, purpose, and time of the meeting. Each Division Chief was asked to then nominate two individuals to attend the meeting. We then directly invited these individuals, asking them to submit one abstract of a current or proposed RCT they were leading. In total, 80 invitations were sent, 52 indicated interest in attending, and 42 people attended the meeting. The agenda and the submitted abstracts are available at http://www.cann-net.ca/images/CSN_2014.pdf.

### Meeting questions

The specific questions posed of meeting attendees were as follows:

1. Should the current CANN-NET clinical trial review and endorsement process continue, and are the ways to increase the value of this review process to investigators?
2. Should we develop a national network and clinical trial infrastructure, and if so, what should be the goals?
3. Should the committee help define a national research clinical trial agenda, and if so, how can this be facilitated?

## Proceedings

RSS opened the meeting and introduced her co-facilitators MW and CR and the other members of the CANN-NET Clinical Trials Committee. All participants then introduced themselves with a brief statement about their motivation for attending. The common themes expressed were a strong enthusiasm for the meeting, a desire to facilitate research at their respective centers, and a desire to improve collaboration across Canadian centers through creation of a formalized trial network.

### a) Current state of CKD, AKI, and dialysis trials in Canada

#### CANN-NET Clinical Trials Committee

The purpose and function of this committee in its current form was summarized by RSS. The CANN-NET Clinical Trials Committee was established in 2010 to review and provide feedback to investigators on their proposed trials. This process of early peer-review from multiple centers is intended to evaluate the significance of the proposed research question(s), improve the methodological quality of the protocol, evaluate and enhance feasibility of recruitment, and ultimately increase funding success of protocols submitted to provincial and national funding agencies. The Committee welcomes trial protocol submissions at any stage of development, and at any time, and it was noted that the review process can be iterative. For investigators seeking a formal letter of support or endorsement to accompany a grant funding application, deadline for receipt of protocols by the committee is 6 weeks before the agency deadline. Each protocol is reviewed independently by two committee members; this is followed by a committee discussion, and constructive written feedback is provided to investigators. If requested, and if in the judgment of the committee the proposed trial is feasible, methodologically sound, and addresses an important clinical question in nephrology (ideally one of the recognized Canadian investigator or patient research priorities [2]), a letter of support or endorsement is provided.

Since 2010, 17 trial protocols have been reviewed, of which 6 have received endorsement from the committee, and 4 have been funded and are in progress [3].The meeting provided an opportunity to reflect on these past activities and to discuss future directions for the committee.

#### Ongoing and planned trials in Canada

Several attendees submitted abstracts of current or proposed clinical trials. Abstracts describing the projects were included in the meeting materials and are posted on the website [4]. These were briefly summarized for the group by each investigator. It was clear that a broad range of clinical trials were ongoing despite lack of a formalized national network. Questions spanned optimal drug therapies, dialysis interventions, and models of delivery of care. Methodologies included parallel and cluster RCTs. Target populations included people with chronic kidney disease, people on dialysis, people with acute kidney injury, and children with kidney diseases. The projects frequently involved collaboration with centers in Canada and internationally. Most trials evinced a “make it happen” philosophy, with resources and funding cobbled together from many sources including start-up funds for new investigators, university awards and grants, as well as tri-council funding.

### b) Patient priorities survey: methods and results

Dr. Manns presented insights from a recent research priority-setting exercise which sought to identify the most important unanswered questions about the management of kidney failure from the perspective of adult patients on or nearing dialysis, their caregivers, and the health care professionals who care for them. With increasing emphasis among health care providers and funders on patient-centered care, this work was an acknowledgement that patients and their caregivers should be included when establishing priorities for research. This thinking is in line with the Canadian Institutes of Health Research (CIHR) Canadian SPOR initiative—“Strategy for Patient-Oriented Research.”

During this process, a national survey of 317 patients, caregivers, and health care providers was conducted to identify unanswered research questions, followed by a facilitated workshop including 34 patients, caregivers, and a multidisciplinary group of health care providers. At this workshop, the top ten research uncertainties were identified which included questions about enhanced communication among patients and providers, dialysis modality options, itching, access to kidney transplantation, heart health, dietary restrictions, depression, and vascular access. A full list is available at http://www.cann-net.ca/committees/research-priorities-survey#results, and more details are available in a recent publication [2].

The group reflected on the contrast between patient and investigator priorities. Although a formal comparison has not yet been performed, the uncertainties of patients appear to focus more on improving symptoms, and optimizing communication, while investigator priorities appeared to focus more on determining how to extend life.

### c) Lessons learned from other successful trial networks

#### Canadian Kidney Transplant Network

Dr. John Gill, president of the Canadian Society of Nephrology, emphasized what he perceived were the key steps in successful development of the Canadian Kidney Transplant Network. He emphasized that they identified key leaders (e.g., division chairs) to advocate for development and prioritization of trial infrastructure at participating sites. Initially, a few key centers were chosen to form the nucleus of the network. Specifically, those centers that had dedicated investigators with proven track records, existing infrastructure in the form of funding and experienced coordinators, and a culture of trial participation among the patients formed the foundation of the network, upon which other centers were added. As the network grew, a formal centrally coordinating site to run multicenter trials was developed. This model has proven extremely successful for them.

#### Australasian Kidney Trials Network

Dr. Meg Jardine described several strategic and tactical lessons learned in the creation and growth of the Australasian Kidney Trials Network (AKTN) [5]. She stressed the importance of trial design, starting with the selection of a research question that resonates strongly with the clinical community. Independent expert advice outside the network and direct stakeholder engagement are sought to set priorities. Simple pragmatic questions and designs are preferred. For example, they specifically avoid long and complex patient selection criteria, requirement for multiple biological samples, and time-consuming measurements—such barriers inhibit trial recruitment, escalate costs, and diminish success and thus are avoided as much as possible. To facilitate recruitment, they collaborate and engage with centers early in the protocol development process, rather than at the end, and they ensure coordination of studies to avoid competition for recruitment in the same patient population. Allowing sufficient time for ethics and institutional review processes at separate sites is a priority. She concluded by emphasizing the human element in networks, the supreme importance of engaging respected opinion leaders, of developing trust based on collaboration and mutual support, and of continually looking outward to recruit, renew, and grow.

#### European Vasculitis Study Group and Canadian Critical Care Trials Group

MW described the structure and practices of the European Vasculitis Study Group (EUVAS) [6] and the Canadian Critical Care Trials Group (CCCTG) [7]. EUVAS is a template for a group of geographically dispersed individuals from a variety of medical backgrounds that meet regularly and undertake multicenter trials without any specific funding as a group. There is an informal structure that includes an executive comprised of about five members, but the group is in most respects egalitarian and inclusive and imposes no membership fees or specific member obligations. Meetings tend to occur alongside major specialty meetings, and members self-fund attendance. The meetings are used to update interested members on the progress of current observational and interventional studies as well as to discuss potential new studies, with an implicit goal of directing the overall field of vasculitis research and avoiding competition between studies. The Canadian Critical Care Trials Group is similar in that it holds regular meetings to which members also self-fund attendance. It differs in that there is an annual membership fee to cover administrative costs and the meetings take place as stand-alone events rather than in conjunction with society meetings. The meetings are a formal venue to discuss proposed projects in detail with feedback from the CCCTG membership. The meetings are also open to non-physician researchers and research staff.

#### Pediatric networks in Canada

Dr. Catherine Morgan described a number of successful pediatric networks in Canada, including the Pediatric Group within the CCCTG and the Pediatric Emergency Research Canada (PERC) [8] network. Both are networks with great success in facilitating high quality pediatric trials. She highlighted that these networks were developed to address the dearth of evidence in pediatric medicine. In a review of randomized trials with interventions of relevance to both adults and children, the effect of the interventions is not studied in children in more than half. She identified that the common threads within successful pediatric networks have been a pre-existing collaborative environment, mentorship and reinforcement from experienced trialists, promotion of a positive culture of research, and provision of access to individuals with trial expertise. She emphasized the value of regular meetings and face-to-face interactions in fostering mentorship and building strategic alliances. Pediatric trials within CCCTG evolved under the mentorship of a strong adult-led group although the network is now very much an integration of pediatric and adult research process. The discussion highlighted that within Canada, there are several barriers to nephrology RCTs in children that could be overcome through the development of a Pediatric Group within an Adult Network, similar to the CCCTG group. Lastly, she discussed StaR Child Health, a research network focused on the agenda of increasing the quality and quantity of pediatric trials. The network is an international group of researchers, methodologists, practitioners, regulators, and journal editors who have created standards (practical and evidence based) in the methodology of pediatric trials. The critical framework provided by this group could be utilized by a pediatric nephrology trial network within Canada.

### d) Small group sessions

After the formal large group presentations, we held a series of small group sessions with the goals of defining the roles and goals of a potential network, defining the role of patient priorities, and determining the most important barriers to conducting nephrology trials in Canada as well as potential solutions.

### Roles and goals of the network and the role of patient priorities

The group was asked to reflect on the themes presented by the previous speakers and address the following three questions:

- What concrete deliverables do you think the clinical trial committee should work towards providing for you?
- What should the specific goals of this network be?
- Should future Canadian nephrology trials focus on patient-identified priorities, and if so, how can we facilitate this?

#### Thematic summary

The discussion was broad and wide ranging, but several recurring themes arose and are summarized below:

1. The CANN-NET clinical trial review process was overwhelmingly thought to be of benefit by the majority. The group felt that this activity should be continued.
2. A formal clinical trial network should be developed. Several potential activities and priorities for this network were discussed. The following goals were felt to be important for the incipient network:
  - To promote a culture of collaboration rather than competition within the Canadian nephrology community.
  - To promote ongoing discussion of clinical trial ideas in order to develop new studies.
  - To identify and address barriers to recruitment for ongoing studies.
  - To hold regular face-to-face meetings of investigators in order to achieve the above three goals. It was felt that regular face-to-face meetings are of critical importance to fostering mentorship and a culture of collaboration (as opposed to competition), and in building and disseminating a culture of research across Canada, and as such was a critical step towards achieving goals a to c. Such meetings would be used for developing trial ideas, scientifically reviewing and improving methodology of existing protocols, discussing ongoing progress and challenges in active trials, conducting post-trial debriefings (e.g., “lessons learned”), and developing knowledge translation strategies (an often neglected initiative).
  - To develop a web-based system to connect investigators with interested centers across Canada. It was felt that a web-based “match-making” system could be created with the following functions:
    i. *A registry of active clinical trials* where centers could log in and identify themselves as interested in participating in a specific study.
    ii. *A registry of centers willing to participate* in trials, including information regarding the following:
      1. Types of patient populations
      2. Specific interests of researchers at that center
      3. Research ethics board processes and contacts
      4. Research coordinator contacts
    iii. *A registry of patients who wish to participate* in trials. This latter point was discussed at length. Such an initiative was thought to be valuable; it involves minimal risk, is not expensive, and ethics approval is not onerous.
  - To develop a central infrastructure to assist investigators with managing clinical trials. Several possible components and functions were discussed, most prominently the following:
    i. *Hiring central coordinator(s)* to assist investigators with identification of centers, training of community health care providers, often nurses, in research coordination, including recruitment and good clinical practice
    ii. *Hiring personnel* to maintain the abovementioned web-based registry of information
    iii. *Consideration of engaging existing Academic Research Organizations (AROs)* to provide this functionality
  - To facilitate and prioritize the funding of national nephrology trials. It was widely felt that the existing activities of protocol review and provision of letters of endorsement were helpful at increasing credibility of trials to national peer-review agencies. However, a formal network should also advocate with government and other funders to increase the pool of funds available. For example, a formal Network could lobby government agencies (e.g., the Ontario Renal Network, British Columbia Provincial Renal Agency) to provide matching funds for CIHR applications approved by the clinical trial network. It was also suggested that the network pursue and sponsor RCT planning grants in collaboration with the Kidney Foundation of Canada (KFoC), to promote study development. In time, through advocacy as well as the conduct of high-quality RCTs in kidney disease, the network would develop a recognized brand as premier facilitator of high-profile research internationally.
3. Patient priorities were discussed. There was a general agreement that patient priorities should be valued and facilitated in Canadian trial research, and that an optimal balance between investigator-driven priorities and patient priorities should be pursued, as these two viewpoints were both necessary and complementary.
4. Patient engagement in research. It was suggested that the process of patient engagement should not be limited to priority-setting exercises and participation in specific trials. Developing a patient-driven culture of research, as exists in other specialties, e.g., oncology, where patients actively seek involvement in RCTs, was suggested as a potential activity. Whether this latter model would work in chronic kidney disease patients who may not perceive their illness as being as severe as oncology patients do is unclear. Currently, advocacy groups such as the Kidney Foundation of Canada are helpful in engaging patients, but patient involvement in research could be further facilitated by the following:
  - Involving patients in the CANN-NET Clinical Trials Committee
  - Involving patients in meetings planning study protocols, expecting that their input would be particularly important in areas including what study outcomes should be included
  - Advertising ongoing trials directly to patients through bulletin boards, websites, and posters rather than waiting for investigators and coordinators to approach them
  - Developing specific initiatives to fund trials addressing patient-identified research priorities
  - Allowing patients to directly join the registry described above
5. Exploiting synergies with the KFoC. Mr. Wim Wolfs, National Director of Research for the KFoC, and Mr. Paul Shay, National Executive Director of the KFoC, suggested that several synergies might be exploited between KFoC and the network. For example, the KFoC has an existing social media network that could be used to improve communication with stakeholders and increase the profile of the network. Private discussion forums could be set up for investigators and CANN-NET groups. Clinical trials could be listed on the website. E-news letters for advertising trials to patients and aiding recruitment could be employed. The KFoC could also fund RCT planning grants and other initiatives supporting trials developed by the network.

### Barriers and potential solutions to conducting nephrology trials in Canada

To facilitate discussion of this topic, small groups were given a hypothetical request to participate in a clinical trial (see Additional file 1 for questions posed) and were asked to reflect on specific barriers and potential solutions to participating in that clinical trial. The groups were asked to approach the problem from two viewpoints: that of a participating center, and that of a principal investigator soliciting participation. The identified barriers and solutions were then shared and discussed with the group. Several recurrent themes emerged from this discussion and are summarized in Table 1.

## Final recommendations

Based on the themes elicited at the meeting, the following action items were endorsed by the CANN-NET Clinical Trials Committee:

1. The committee will proceed with the formation of a Canadian clinical trial network, planning twice-yearly meetings of network participants to be held initially in conjunction with national or international society meetings.
2. The committee will engage with the Kidney Foundation of Canada and other agencies as needed to develop online networking tools as described.
3. The committee will engage with the Kidney Foundation of Canada to launch a new funding competition for meeting and planning grants with the intent of providing financial support for meetings that will enable the conduct of prospective research (i.e., randomized controlled trials or prospective cohort studies).
4. The committee will further consider ways in which a core RCT infrastructure might be funded and maintained.
5. The committee will advocate for a more patient-centered approach to nephrology research by supporting studies that include patient- and/or caregiver-identified priorities.

## Additional file

Additional file 1:
**Agenda and list of questions discussed.**
