# Supplementary material for: Establishing a Canadian national clinical trials network for kidney disease: proceedings of a planning workshop
Source: Can J Kidney Health Dis. 2015 Nov 17;2:46. doi: 10.1186/s40697-015-0080-7 (PMC4650502; doi:10.1186/s40697-015-0080-7)
Supplement: Additional file 1: — Agenda and list of questions discussed. [file 40697_2015_80_MOESM1_ESM.pdf]

CANN•NET
Canadian Kidney
Knowledge Translation
And Generation Network

CANADIAN CLINICAL TRIAL NETWORK

Date: April 24, 2014
Time: 8:00 am – 11:30 am
Location: Renaissance Hotel-Ballroom

Objectives:

1. To discuss the current state of CKD, AKI, and dialysis clinical trials in Canada
2. To discuss the current and future role of the CANN-NET Clinical Trials Committee
3. To initiate the development of a collaborative Canadian Nephrology Clinical Trials Network, through meeting of investigators, identification of barriers, and discussion of solutions

Agenda:

Item	Presenter	Time
**1. Introduction**		
a) Welcome and Housekeeping Issues	Rita Suri	8:00
b) Purpose of the Meeting in context of CANN-NET	Braden Manns	8:05
**c)** Self-Introductions	Group	8:10
**2. Current state of CKD, AKI and dialysis trails in Canada:**		
a) CANN-NET Clinical Trials review process	Rita Suri	8:20
b) Ongoing and planned trials in Canada	Group	8:25
c) Patient priorities survey	Braden Manns	8:45
d) Discussion:	Rigatto/Suri/Group	8:55

I. What did you hope to gain by coming to this meeting?
II. What concrete deliverables do you think that the Clinical Trials Committee should work towards providing for you?
III. What should the specific goals of this network be?
e.g. To have infrastructure and money to give people such as small planning grants ($15K each), access to a national research coordinator, etc., OR to be a loose network providing knowledge of what is going on and access to people.
IV. Should future Canadian nephrology trials focus on patient-identified priorities, and if so, how can we facilitate this?

Item	Presenter	Time
Break		**9:30**
**3. Development of a Canadian Nephrology Clinical Trials Network**		
a) Examples of successful networks:		9:45
• Australian Kidney Trials Network	Meg Jardine	
• Canadian Kidney Transplant Network	John Gill	
• Other - UK EUVAS, CCCTG	Mike Walsh	
• Pediatric Networks	Susan Samuel	
b) Discussion	Walsh/Suri/Group	10:05

I. What are the barriers and potential solutions to participating in nephrology trials in Canada?
II. What specific steps can this committee and network take to implement solutions

Item	Presenter	Time
**4. Wrap Up, Discussion of Next Steps:**	Suri/Walsh	11:05

Attachment for Agenda Item 4b:

Assuming you feel the topic is worthwhile and will provide important information,
for the two problems below, please discuss the specific barriers and potential solutions with respect to conducting trials in dialysis and chronic kidney disease at your centre.

PROBLEM 1:	*PROBLEM 2:*
Dr. Smith is conducting a randomized trial of blood pressure lowering algorithm strategies in hemodialysis patients, including pharmacotherapy, and reduction of target weight. The follow-up is 2 years. He has invited you to enroll and randomize 25 patients over 1 year. Do you agree to participate? Why or why not?	Dr. LeBlanc is conducting a randomized trial of a pre-dialysis education strategy to increase uptake of arteriovenous fistulae and home dialysis. She has invited you to participate and enroll 50 patients over 1 year. Do you agree to participate? Why or why not?

Guiding Questions:

1. From an investigator standpoint: What are the barriers to recruiting additional centers?
2. From a participating site standpoint: What are the specific barriers to participating in clinical trials at your center? (consider logistical, financial, time, knowledge, or other)

Try to divide these into easily surmountable, surmountable with some effort or money, likely insurmountable

3. Are there potential specific solutions that could be implemented at your center?

Try to divide these into local solutions, solutions that would potentially be solved through having a national Clinical Trials Committee infrastructure

Examples:

Barrier:
- We have no research coordinator.
- Surmountable with some effort and money.

Solution:
- Hire a nurse with dialysis experience part-time.
- Solution would be facilitated by CANN-NET Clinical Trials Committee infrastructure if specific training of the nurse could be provided by them.
